# Degradation kinetics of Ti-Cu compound layer in transient liquid phase bonded graphite/copper joints

**DOI:** 10.1038/s41598-018-33446-3

**Published:** 2018-10-12

**Authors:** Jincheng Lin, Mei Huang, Weiqi Yang, Lili Xing

**Affiliations:** Sino-French Institute of Nuclear Engineering and Technology, Sun Yat-sen University, Zhuhai, 519082 P. R. China

## Abstract

The continuous Ti-Cu compound layer produced in brazing of graphite to copper with Ti foil is found to be seriously detrimental to joint properties due to its brittleness. In this work, a transient liquid phase (TLP) bonding method with a diffusion process below melting point is developed to realize a Ti-Cu compound layer free joint. The degradation of Ti-Cu compound layer depends on two simultaneously occurring processes, namely flow of titanium atoms to copper substrate and that to TiC layer on graphite. The latter is determined by growth kinetics of TiC layer based on carbon diffusion process. A degradation model is proposed and applied to optimize the TLP bonding. The improved graphite/copper joints without Ti-Cu compound layer show 20.8% higher in shear strength compared with that of brazing joints.

## Introduction

Carbon based materials (CBMs) including graphite and carbon fiber reinforced carbon composites have been widely used as structural materials and electronic components owing to their unique combination of high melting point, high thermal/electrical conductivity, excellent thermal fatigue and plasma compatibility^1–4^. In most practical applications, CBMs requires joining with metal, especially copper or copper alloy, to form complete structures with desirable and unique characteristics such as resistance to physical/radiation damage, abrasion resistance, higher strength and toughness^5^. For example, the fabrication of divertor in ITER involves joining of plasma facing materials (PFMs, including CBMs and tungsten) to copper based heat sink^6^. In electromechanical industry, the commutator integrated by graphite and copper fittings has high lubrication and abrasion resistance. The quality of graphite/copper joint is an important indicator to the performance and service life of D.C. motor^7,8^.

For joining of CBMs to copper, brazing is the most commonly used technique due to its simplicity, low cost and good adaptability to different joint shapes. Because copper is inert to carbon characterized by neither with mutual solubility nor formation of stable carbides^9^, some strong carbide-forming element like titanium is usually added in filler alloy to facilitate reactive wetting. In the past decade, various active brazing alloys (such as Cu50TiH_2_^10^, Cu-ABA^11^, TiCuNi^6^) have been used to join CBMs and copper. For these brazing alloys, the content of Ti is a key factor for joint quality. Generally, Ti concentration is inversely proportional to contact angles of liquid alloy. It’s reported that the wetting on CBMs requires the addition of Ti at least 10 at.% so as to offset the influences of pre-oxidation or possible pollution on substrate surface^9^. Nevertheless, excessive Ti may lead to abundant intermetallic compounds (IMCs) in seam. Since residual stress caused by the mismatch of coefficient of thermal expansion (CTE) between CBMs and copper is easily created in the joint, the brittle IMCs dramatically increase the possibility of joint failure in service^12–14^. Thus, how to solve the conflict between wetting and IMCs-forming tendency is essential to the property improvement of CBMs/copper joints.

In this work, the graphite is successfully joined to copper by improved transient liquid phase (TLP) bonding with Ti interlayer. It’s known that typical TLP bonding involves long isothermal solidification process at joining temperature to make sufficient diffusion of the interlayer element (or a constituent of an alloy interlayer) into substrate materials^15,16^. But in our method, the contact reaction between Ti and Cu is first carried out at 920 °C to promote wetting, then isothermal process (at 860 °C) is set below the melting point so as to prevent sever infiltration of liquid alloy into graphite. In the solid isothermal process, Ti-Cu IMCs produced in the contact reaction can dissolve by two ways: the first is diffusion of Ti to Cu substrate and the second is competing reactions with TiC layer at graphite interface. The degradation of Ti-Cu IMCs is governed both by Ti diffusion behavior and TiC growth kinetics. The degradation model proposed by this work can be used to predict the residual IMCs layer in seam and is helpful to improve the joining technique.

This paper is divided into three parts. In the first one, the microstructure of joint after contact reaction is characterized and the brittle IMCs layer is identified. In the second part, the experimental results as well as calculations on degradation kinetics of Ti-Cu IMCs layer are shown and discussed. In the third part, the microstructure and mechanical properties of graphite/copper joint joined by an optimized TLP bonding are presented.

## Results and Discussion

### Microstructure of graphite/Cu joint brazed with Ti interlayer

The graphite/Cu brazing involves a series of interfacial reactions in seam. During heating, solid-state diffusion reactions occur first between Ti interlayer and Cu substrate, producing a small quantity of Ti-Cu compounds, such as TiCu, Ti_3_Cu_4_ and TiCu_4_. As the temperature rises above the eutectic point (875 °C), the Cu-Ti eutectic liquid forms and fills the gap between graphite and Cu by capillarity. The wetting process on graphite accompanies fast growing of TiC layer at graphite/liquid interface. Besides, mutual diffusion between Ti and Cu results in enrichment of Cu in Cu-Ti liquid. So in cooling process, the peritectic reaction (Liquid + Cu → TiCu_4_) happens and a continuous TiCu_4_ layer forms in the joint.

**Figure 1:**
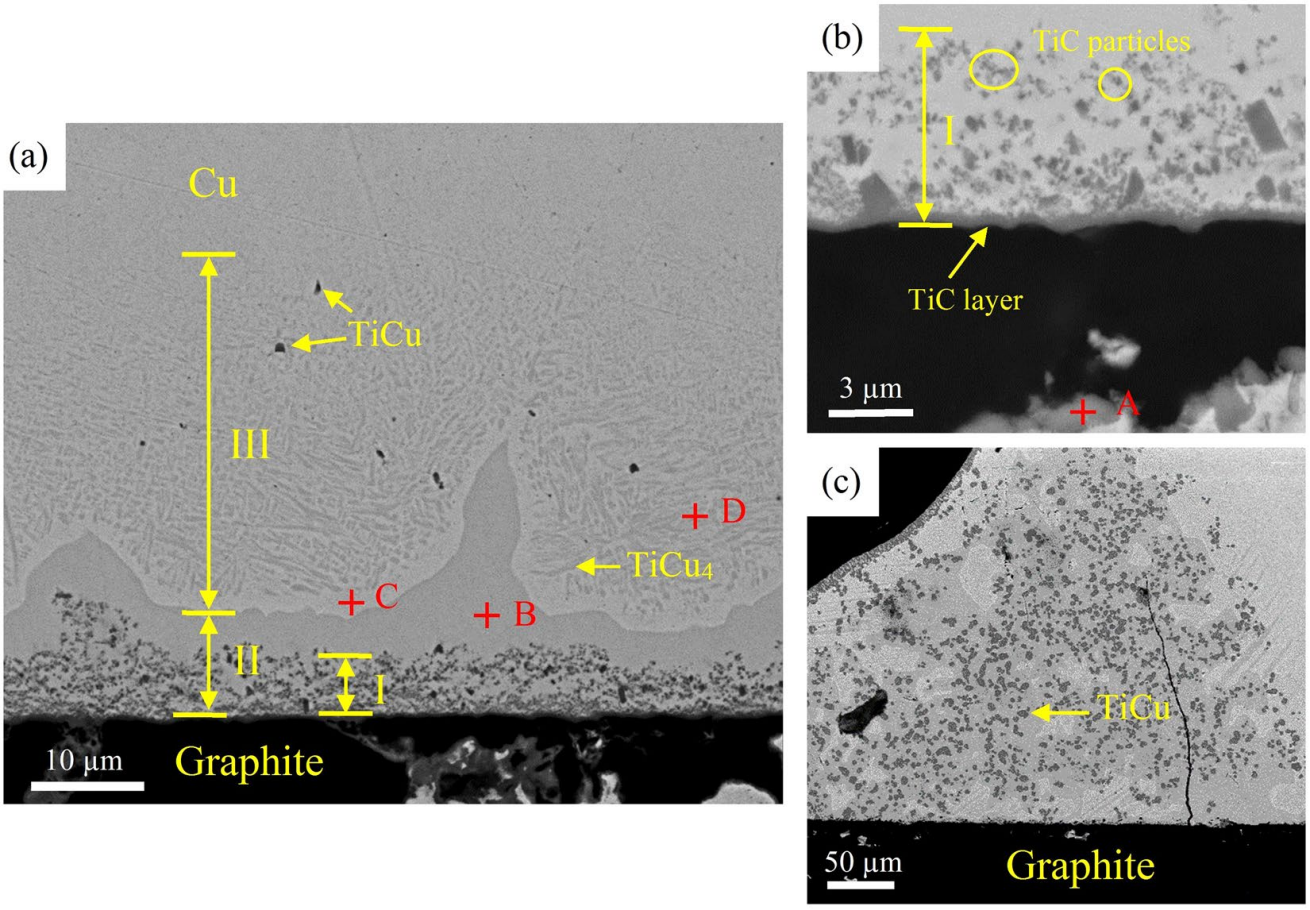
Backscattered electron micrographs of graphite/Ti (50 μm)/Cu joint brazed at 920 °C for 10 min. (**a**) Microstructure of the joint. (**b**) Interfacial reaction layer on graphite. (**c**) Fillet of the joint.

**Figure 2:**
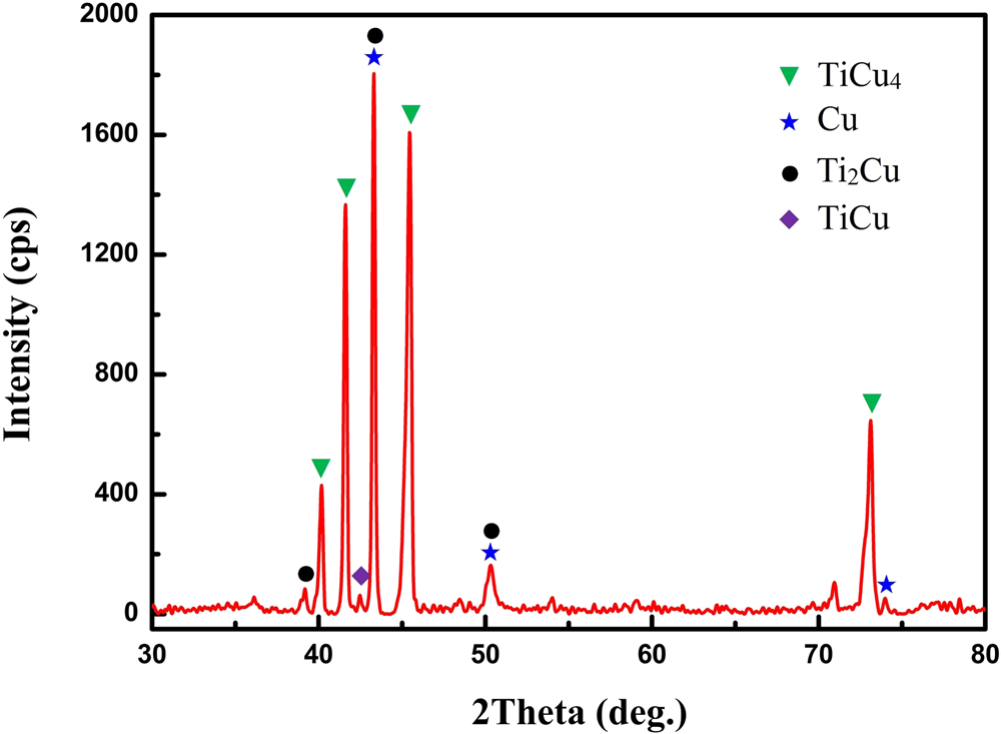
XRD pattern of graphite/Ti (50 μm)/Cu joint plane.

Figure 1 shows the microstructure of graphite/Cu joint brazed at 920 °C for 10 min. Three regions can be distinguished in the joint. Region I is comprised by a thin TiC layer (point A in Table 1) and nanoparticle layer (as shown in Fig. 1b). It’s inferred small amount of carbon atoms diffuse through TiC layer and dissolve into the Cu-Ti liquid. Subsequently, TiC particles precipitate out of the liquid. Region II is the continuous TiCu_4_ layer (point B) produced by peritectic reaction. Region III is the transition layer from brazing liquid to Cu substrate. As the solubility of Ti in Cu significantly decreases during cooling, lath like TiCu_4_ phases precipitate from the supersaturate Cu_ss_ (point C) to form this two-phase region (TiCu_4_ + Cu_ss_, point D). It is noticed that some dark TiCu blocks are also found in this layer. As mentioned above, various Ti-Cu compounds are produced by solid-state reactions during heating. But after the melting of Ti interlayer, majority Ti-Cu compounds dissolve in the liquid except for the thermodynamically stable TiCu (∆G = −17530 + 3.37 T J/mol^17^). Although most TiCu blocks can be extruded to the fillet (as shown in Fig. 1c) by liquid flow, small amount of TiCu are still left in the seam. Figure 2 is the XRD patterns of joint plane prepared by polishing layer by layer. It confirms the existence of TiCu_4_ and TiCu.

**Table 1:** EDS analysis of the zones identified in Fig. 1.

Points	Elements (at.%)			Possible Phase
	Cu	Ti	C	
A	3	42	55	TiC
B	79	21	—	TiCu_4_
C	97	3	—	Cu_ss_
D	90	10	—	TiCu_4_ + Cu_ss_

**Figure 3:**
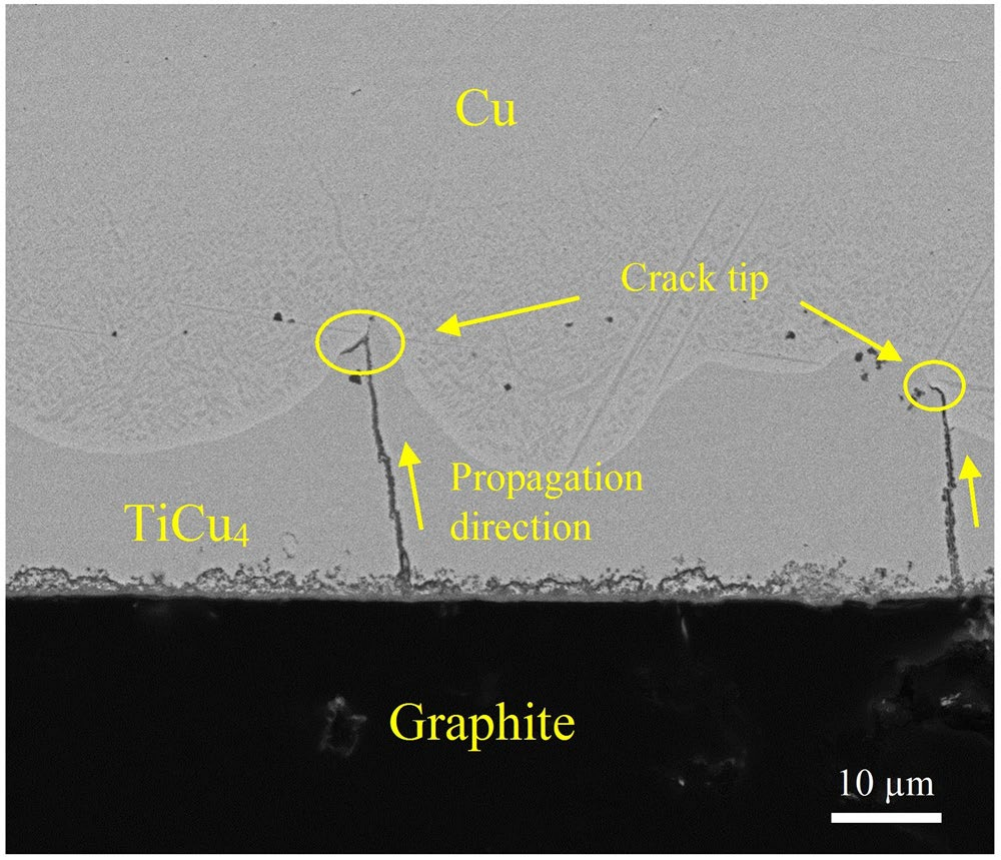
Microstructure of graphite/Ti (50 μm)/Cu joint after one week’s standing without any load.

As indicated in Fig. 1a, it seems that the joints brazed with Ti interlayer are metallurgically sound and defect free. However, after one week’s standing without any load, cracks were widely found in all samples as shown in Fig. 3. These cracks initiate in graphite interface, propagate vertically through TiCu_4_ layer and stop in Cu_ss_ in region III. Obviously, the residual stress resulted from large mismatch of CTE between the two dissimilar substrates (*α*_Cu_ = 16.5 × 10^−6^ K^−1^, *α*_graphite_ = 3.9 × 10^−6^ K^−1^) is the main reason for cracking. In graphite/Cu joints, Cu will be under tension and the graphite under compression during cooling from brazing temperature^18^. The elastic residual stress in Cu substrate, roughly estimated by *σ* = *E*Δ*α*Δ*T* (*E*: Young’s module, Δ*α*: CTE mismatch, Δ*T*: temperature interval)^19^, is 1416 MPa, which exceeds the yield strength of Cu. In region III of the seam, stress can be released by deformation of Cu_ss_. But in region II, fracture rather than plastic deformation is more likely to happen in the brittle Ti-Cu compounds. This hidden danger may lead to a catastrophic consequence when the joint serves in alternating stress environments. Thus, how to eliminate the Ti-Cu compounds, especially the thick continuous TiCu_4_ layer, is a key issue in practical application.

### Degradation kinetics of Ti-Cu compound layer in TLP bonding

**Figure 4:**
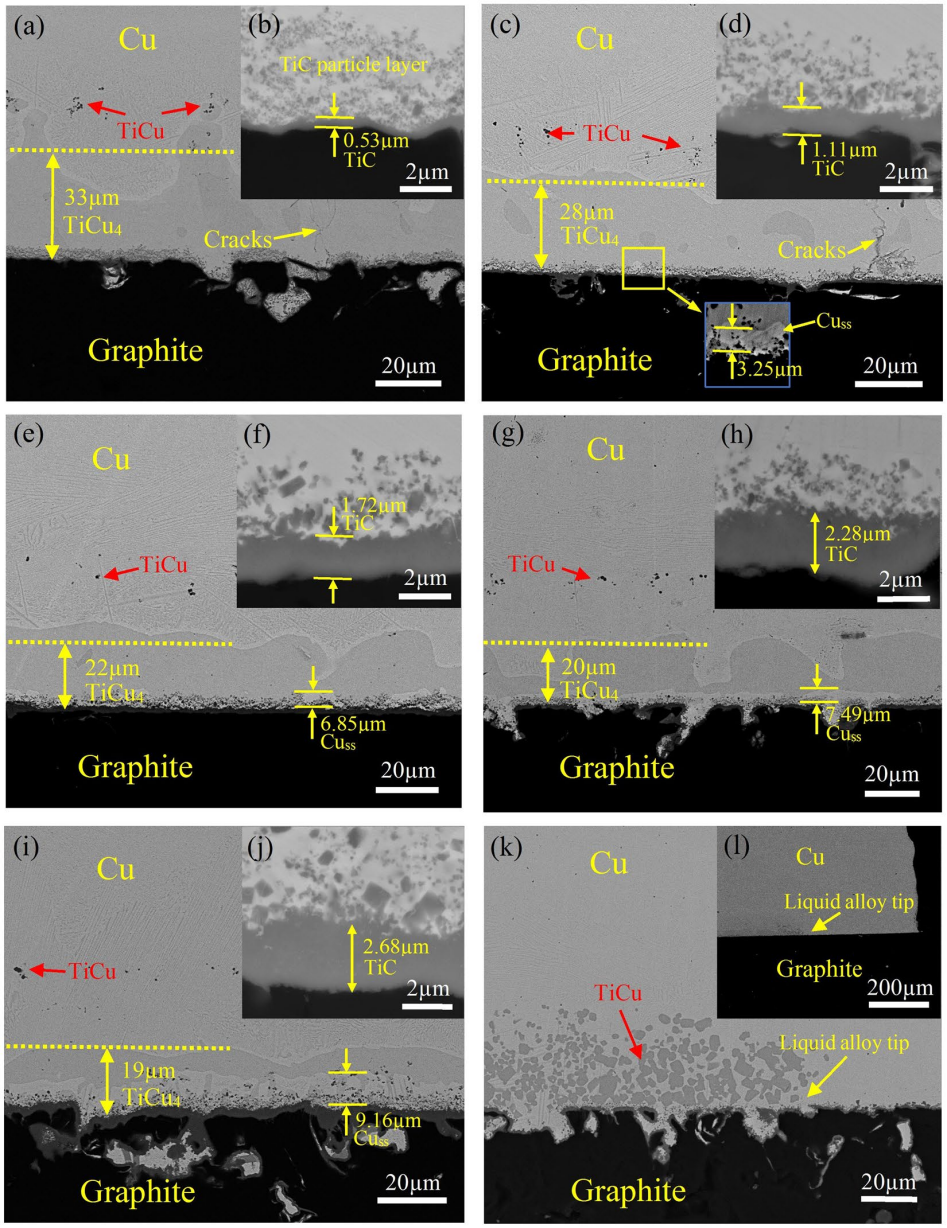
Backscattered electron micrographs of graphite/Ti (4 mm × 4 mm × 20 μm)/Cu joints prepared by TLP bonding with various holding time at 860 °C. (**a**) 0 h. (**b**) Interfacial region of (**a**). (**c**) 1 h. (**d**) Interfacial region of (**c**). (**e**) 3 h. (**f**) Interfacial region of (**e**). (**g**) 4.5 h. (**h**) Interfacial region of (**g**). (i) 6 h. (**j**) Interfacial region of (**i**). (**k**,**l**) Boundary of the joint shown in (**a**).

In order to realize a Ti-Cu compound layer free joint, TLP bonding approach is proposed and the degradation of Ti-Cu compound layer is studied by using a smaller 4 mm × 4 mm × 20 μm titanium interlayer. Figure 4 shows the microstructure of graphite/Cu joints with solid diffusion times of 0, 1, 3, 4.5 and 6 hours. It is observed that TiCu compounds formed before melting are nearly at the same distance to graphite interface regardless of variation of holding time. The stable positions of TiCu reflect the low fluidity of brazing melt. Besides, Fig. 4k and l show that the Ti-Cu melts are trapped in the seam and no extrusion happens. In this condition, the continuous TiCu_4_ layer just after melting is around 34 μm thick. As holding time extended, the thickness of TiCu_4_ layer decreases while the thickness of region III (Cu_ss_ + TiCu_4_) increases. Apparently, the diffusion of Ti to Cu substrate at high temperature consumes TiCu_4_ and forms a supersaturated solid solution region in Cu substrate. Another way leading to the reduction of TiCu_4_ layer is the growth of TiC on graphite. As TiC is far more thermodynamically stable than TiCu_4_ (ΔG_TiCu4_ = −7600 + 3.12 T J/mol^20^), the Ti atoms required for growth of TiC nearly all come from dissolving of TiCu_4_ (TiCu_4_ → Ti + 4Cu). Figure 4 shows that, with extension of holding time, the thickness of TiC layer gradually increases while the boundary of TiCu_4_ layer recedes, leaving a narrow Cu_ss_ layer as a decomposition product between TiCu_4_ and TiC.

In order to quantitatively predict the dissolving rate of TiCu_4_ layer, a diffusion model is developed in terms of error function solutions for Fick’s second law. The present joint involves three interfaces at the beginning: graphite/TiC interface, TiC/TiCu_4_ interface and TiCu_4_/Cu_ss_ interface. The diffusion of Ti into Cu substrate and growth of TiC layer can both lead to dissolving of TiCu_4_ layer. But considering the two processes occur independently at different sides of TiCu_4_ layer, the quantity of TiCu_4_ consumed on the side of Cu substrate is evaluated by measuring from the graphite/TiC interface to TiCu_4_/Cu_ss_ interface (as shown in Table 2).

**Table 2:** The thickness of TiCu_4_ and TiC layer in graphite/copper joints.

Diffusion times at 860 °C	TiCu_4_ layer thickness (μm)^*^		TiC layer thickness (μm)	
	Average	SD	Average	SD
0 h	32.86	9.26	0.53	0.05
1 h	27.92	2.42	1.11	0.22
3 h	21.98	3.62	1.72	0.23
4.5 h	20.11	6.86	2.28	0.25
6 h	19.03	2.71	2.68	0.32

*Measuring from the graphite/TiC interface to TiCu_4_/Cuss interface.

The Fick’s second law of diffusion, relating the changes in concentration of Ti with time and location is:

1 $$\frac{\partial c}{\partial t}=D\frac{{\partial }^{2}c}{\partial {x}^{2}}$$

where *D* is the diffusion coefficient, *c* is the concentration, *t* is diffusion time and *x* is distance. The copper substrate is considered as semi-infinite medium and the TiCu_4_/Cu_ss_ interface is treated as the not-moving planar boundary. In Ti-Cu system, TiCu_4_ has a narrow stoichiometric range. Thus, at the TiCu_4_/Cu_ss_ boundary, the Ti fraction in TiCu_4_ is assumed to have the lowest value based on Ti-Cu binary diagram^21^ and the solid solubility of Ti in Cu is saturated (6.5 at.%) at 860 °C. Due to the small concentration difference of Ti in Cu, it is assumed that the diffusion coefficient of Ti is independent of the concentration in Cu substrate. With these assumptions, the initial and boundary conditions can be written as:

Initial condition (*t* = 0)

2 $$c(t=0,\,x\ge 0)=0$$

Boundary conditions (*t* > 0)

3 $$c(t > 0,\,x=0)={c}_{{\rm{Ti}},{\rm{\max }}}^{{{\rm{Cu}}}_{{\rm{ss}}}}$$

4 $$c(t > 0,\,x=\infty )=0$$

Here ${c}_{{\rm{Ti}},{\rm{\max }}}^{{{\rm{Cu}}}_{{\rm{ss}}}}$ and *c* represent Ti atomic concentration (atoms/cm^3^) in Cu_ss_ at TiCu_4_/Cu_ss_ interface and Cu substrate, respectively. The concentration of Ti could be converted from its atomic fraction (*x*) by

5 $$c=\frac{x\rho {\rm{n}}{N}_{A}}{M}$$

Where *ρ* and *M* are density and molar mass of matrix, respectively. *N*_*A*_ is Avogadro constant (*N*_*A*_ = 6.022 × 10^23^) and *n* is the atom number per matrix molecule. In copper solid solution, *ρ* and *M* are approximate to that of pure Cu (*ρ* = 8.93 g/cm^3^)^22^. So the Ti concentration in saturated Cu_ss_ (6.5 at.% Ti) is calculated as ${c}_{{\rm{Ti}},{\rm{\max }}}^{{{\rm{Cu}}}_{{\rm{ss}}}}$ = 5.46 × 10^21^ atom/cm^3^. The solution for Ti concentration in Cu satisfying Eqs (1–4) can be written as:

6 $$c(x,\,t)={c}_{{\rm{Ti}},{\rm{\max }}}^{{{\rm{Cu}}}_{{\rm{ss}}}}[1-{\rm{erf}}(\frac{x}{2\sqrt{{D}_{{\rm{Ti}}}^{{\rm{Cu}}}t}})]$$

${D}_{{\rm{Ti}}}^{{\rm{Cu}}}$ is the titanium diffusion coefficient in copper. So the flow of Ti atoms (*J*_Ti_) passing though TiCu_4_/Cu interface can be given as

7 $${J}_{{\rm{Ti}}}(x=0)=-\,{D}_{{\rm{Ti}}}^{{\rm{Cu}}}\frac{\partial c(x=0,\,t)}{\partial x}={c}_{{\rm{Ti}},{\rm{\max }}}^{{{\rm{Cu}}}_{{\rm{ss}}}}\sqrt{\frac{{D}_{{\rm{Ti}}}^{{\rm{Cu}}}}{\pi t}}$$

In isothermal process, it is assumed that Ti atoms from dissolved TiCu_4_ all diffuse into Cu substrate. Applying the rule of atom conservation at the interface, one can write,

8 $$({c}_{{\rm{Ti}},{\rm{\min }}}^{{{\rm{TiCu}}}_{4}}-{c}_{{\rm{Ti}},{\rm{\max }}}^{{{\rm{Cu}}}_{{\rm{ss}}}})\frac{dw}{dt}=-\,{J}_{{\rm{Ti}}}(x=0)$$

where *w* represents the thickness of TiCu_4_ layer (by measuring from the graphite/TiC interface to TiCu_4_/Cu_ss_ interface).

The concentration of Ti in TiCu_4_ at the boundary is considered as the lowest (${x}_{{\rm{Ti}},{\rm{\min }}}^{{{\rm{TiCu}}}_{{\rm{4}}}}=0.194$) in its stoichiometric range. According to Eq. (5), ${c}_{{\rm{Ti}},{\rm{\min }}}^{{{\rm{TiCu}}}_{{\rm{4}}}}$ is 1.50 × 10^22^ atoms/cm^3^ using the density from ref.^22^. By integration of Eq. (8), the thickness of TiCu_4_ layer (*w*) can be expressed in terms of time (*t*),

9 $$w={w}_{0}-2\frac{{c}_{{\rm{Ti}},{\rm{\max }}}^{{{\rm{Cu}}}_{{\rm{ss}}}}}{{c}_{{\rm{Ti}},{\rm{\min }}}^{{{\rm{TiCu}}}_{{\rm{4}}}}-{c}_{{\rm{Ti}},{\rm{\max }}}^{{{\rm{Cu}}}_{{\rm{ss}}}}}\sqrt{\frac{{D}_{{\rm{Ti}}}^{{\rm{Cu}}}}{\pi }t}$$

*w*_0_ is the initial thickness of TiCu_4_ layer. Equation (9) indicates the average thickness of the TiCu_4_ decreases linearly with the square root of time. Applying the experimental values of Table 2, the variation of TiCu_4_ thickness can be expressed as,

10 $$w=33.69-6.12\sqrt{t}\,(t\,{\rm{in}}\,{\rm{hour}})$$

**Figure 5:**
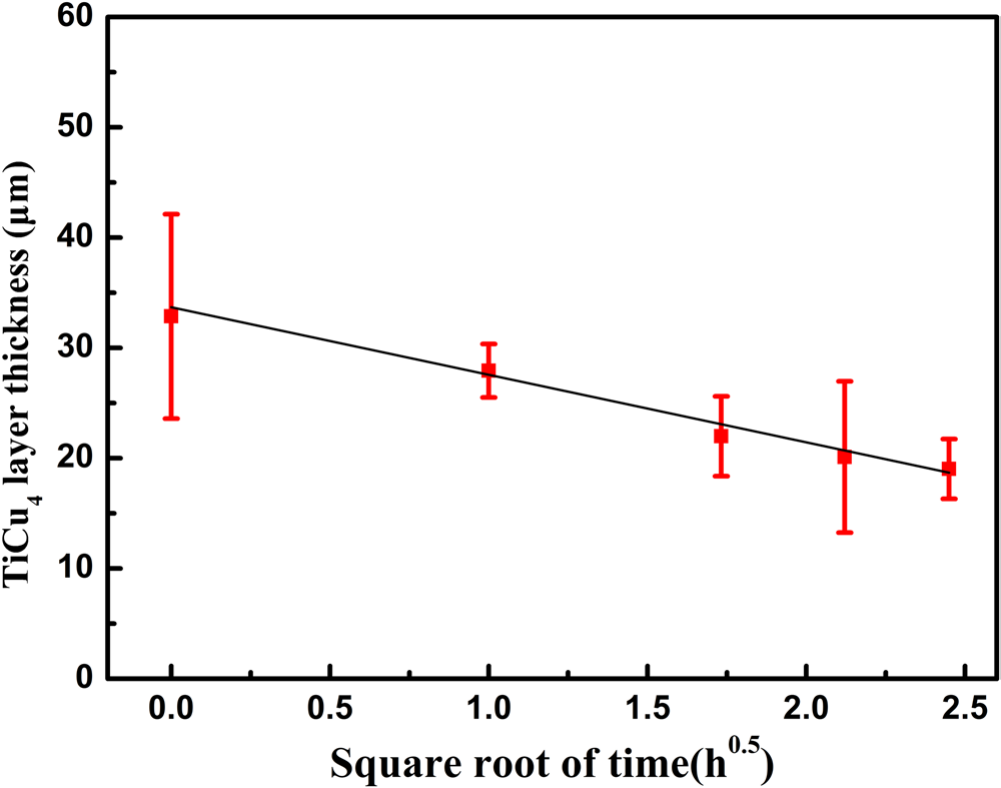
TiCu_4_ layer thickness as a function of holding time at 860 °C.

The fitting curve (Fig. 5) exhibits high correlation (*R*^2^ = 0.974) and gives the titanium diffusion coefficient in copper as ${D}_{{\rm{Ti}}}^{{\rm{Cu}}}=2.49\times {10}^{-14}\,{m}^{2}/s$. From dynamics, the dissolving of TiCu_4_ layer involves two processes including decomposing reaction (TiCu_4_ → Ti + 4Cu) and atomic diffusion. But the calculated ${D}_{{\rm{Ti}}}^{{\rm{Cu}}}$ in this work is quite similar to the result (6.37 × 10^−14^ m^2^/s) obtained from a pure Ti/Cu diffusion couples^23^. This indicates that the kinetics of TiCu_4_ decomposing reaction is quite fast, which is possible related to the poor thermodynamic stability of TiCu_4_.

**Figure 6:**
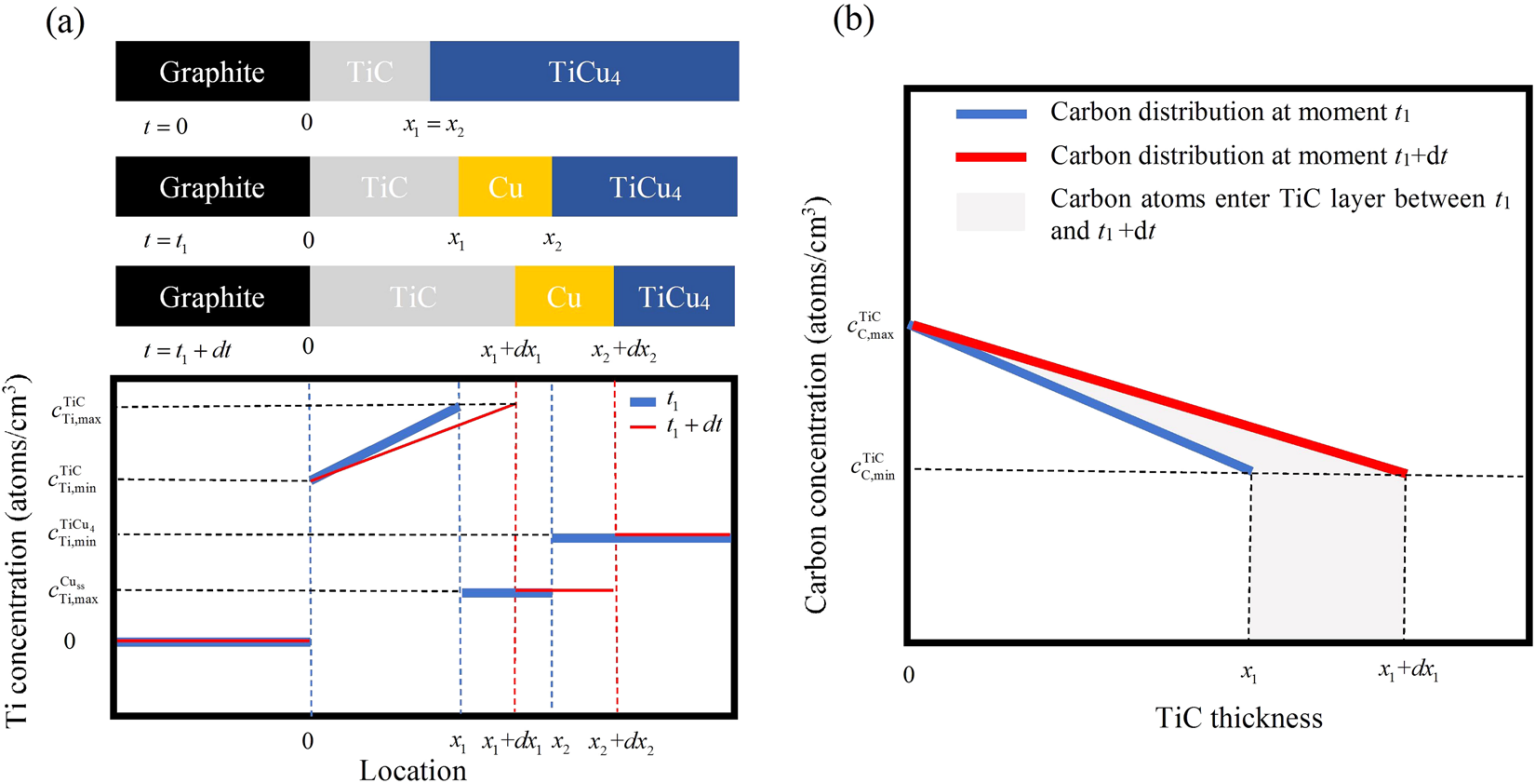
(**a**) Schematic of reaction layers formed during Ti diffusion in Cu_ss_. (**b**) Carbon concentration distribution in TiC layer from *t*_1_ to *t*_1_ + *dt*.

The growth of TiC layer on graphite is considered as another independent process consuming TiCu_4_ in seam. Figure 4 shows that decomposing of TiCu_4_ on graphite side leads to a narrow Cu_ss_ layer on TiC interface. According to ref.^24^ and calculation in this work, the ${D}_{{\rm{Ti}}}^{{\rm{Cu}}}$ is much higher than the diffusion coefficient of carbon in TiC $({D}_{{\rm{C}}}^{{\rm{TiC}}}=12.74\times {10}^{-16}\,{{\rm{m}}}^{2}/{\rm{s}})$^24^, indicating that the Ti flux in Cu_ss_ may satisfy the growth of TiC. Therefore it’s assumed the TiC boundary adjacent to Cu_ss_ and all the Cu_ss_ layer have saturated Ti in their phases. The concentration difference within TiC layer can be considered to represent the average compositional gradient in this phase. Figure 6a shows the Ti concentration profile of this reaction system. In this figure, *x*_1_ and *x*_2_ stand for the location of TiC/Cu_ss_ boundary and Cu_ss_/TiCu_4_ boundary, respectively. At the beginning of solid diffusion (*t* = 0), TiCu_4_ layer directly contacts with TiC, the thickness of Cu_ss_ is zero, thus *x*_1_ = *x*_2_. At time *t* = *t*_1_, TiCu_4_ layer degrades to location *x*_2_ while TiC layer grows to location *x*_1_. The decomposing rate of TiCu_4_ on graphite side can be derived from the growth rate of TiC. Previous investigations have proved that the diffusion coefficient of carbon is far more higher than that of titanium in TiC^24^. So in this case, it’s assumed the growth of TiC layer is dominant by carbon diffusion from graphite interface to TiC/TiCu_4_ (or Cu_ss_) interface. Additionally, the diffusion of carbon in TiCu_4_ and Cu_ss_ is neglected due to the extremely limited solubility of carbon in the two phases. The initial and boundary conditions can be written as:

Initial condition (*t* = 0)

11 $${x}_{{\rm{1}}}={x}_{2}$$

Boundary conditions (*t* ≥ 0)

12 $$c(t,\,x=0)={c}_{{\rm{C}},\max }^{{\rm{TiC}}}$$

13 $$c(t,\,x={x}_{1}(t))={c}_{{\rm{C}},{\rm{\min }}}^{{\rm{TiC}}}$$

14 $${J}_{{\rm{C}}}(t,\,x=0)={D}_{{\rm{C}}}^{{\rm{TiC}}}\frac{{c}_{{\rm{C}},{\rm{\max }}}^{{\rm{TiC}}}-{c}_{{\rm{C}},{\rm{\min }}}^{{\rm{TiC}}}}{{x}_{{\rm{1}}}(t)}$$

Here ${c}_{{\rm{C}},{\rm{\max }}}^{{\rm{TiC}}}$ and ${c}_{{\rm{C}},{\rm{\min }}}^{{\rm{TiC}}}$ are the maximum (48.5 at.%, 4.81 × 10^22^ atoms/cm^3^) and minimum (38.8 at.%, 3.85 × 10^22^ atoms/cm^3^) carbon atom concentration in TiC at 860 °C according to Ti-C binary diagram^21^ and TiC density (*ρ*_TiC_ = 4.93 g/cm^3^)^24^. Figure 6b shows the changing of carbon concentration in TiC within time *dt*. By using conservation of carbon atoms, the TiC growth can be given as:

15 $$\frac{{c}_{{\rm{C}},{\rm{\max }}}^{{\rm{TiC}}}-{c}_{{\rm{C}},{\rm{\min }}}^{{\rm{TiC}}}}{2}d{x}_{{\rm{1}}}+{c}_{{\rm{C}},{\rm{\min }}}^{{\rm{TiC}}}d{x}_{1}={D}_{{\rm{C}}}^{{\rm{TiC}}}\frac{{c}_{{\rm{C}},{\rm{\max }}}^{{\rm{TiC}}}-{c}_{{\rm{C}},{\rm{\min }}}^{{\rm{TiC}}}}{{x}_{{\rm{1}}}}dt$$

Rearranging Eq. (15)

16 $${x}_{{\rm{1}}}d{x}_{{\rm{1}}}=2{D}_{{\rm{C}}}^{{\rm{TiC}}}\frac{{c}_{{\rm{C}},{\rm{\max }}}^{{\rm{TiC}}}-{c}_{{\rm{C}},{\rm{\min }}}^{{\rm{TiC}}}}{{c}_{{\rm{C}},{\rm{\max }}}^{{\rm{TiC}}}+{c}_{{\rm{C}},{\rm{\min }}}^{{\rm{TiC}}}}dt$$

The expression of TiC layer front location (*x*_1_) as a function of time can be obtained by integration of Eq. (16) with an initial position *x*_1_(*t* = 0) = 0.53 µm (Table 2).

17 $${x}_{{\rm{1}}}=\sqrt{4{D}_{{\rm{C}}}^{{\rm{TiC}}}\frac{{c}_{{\rm{C}},{\rm{\max }}}^{{\rm{TiC}}}-{c}_{{\rm{C}},{\rm{\min }}}^{{\rm{TiC}}}}{{c}_{{\rm{C}},{\rm{\max }}}^{{\rm{TiC}}}+{c}_{{\rm{C}},{\rm{\min }}}^{{\rm{TiC}}}}t+{({x}_{1}(t=0))}^{2}}$$

**Figure 7:**
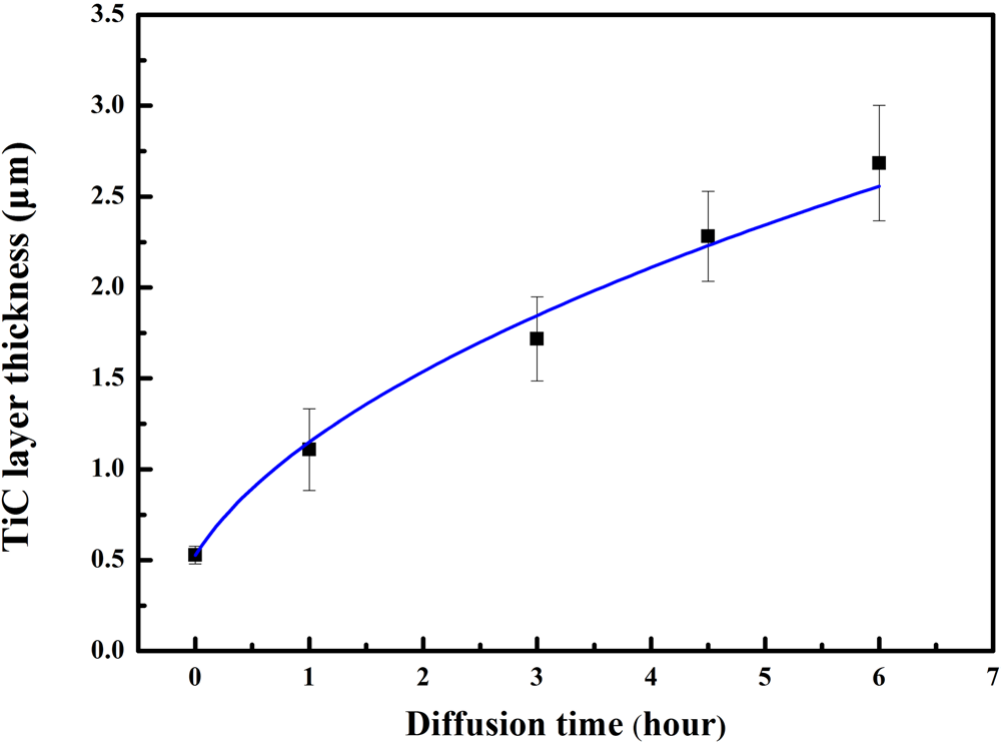
TiC layer thickness as a function of holding time at 860 °C.

By fitting the experimental results shown in Fig. 7 to Eq. (17), it’s obtained that

18 $${x}_{1}=\sqrt{1.046t+0.277}\,t\,{\rm{in}}\,{\rm{hour}}\,({R}^{2}=0.994)$$

The diffusion coefficient of carbon in TiC $({D}_{{\rm{C}}}^{{\rm{TiC}}})$ is given as 6.54 × 10^−16^ m^2^/s. The result in this work is similar to the reported value 12.74 × 10^−16^ m^2^/s at 860 °C^24^. The small deviation of the two values may come from the neglect of carbon diffusion in Cu_ss_ in this work, since small quantity of nano TiC particles indeed precipitate nearby the continuous TiC layer, which is difficult to be taken into the thickness of TiC layer equivalently.

Based on the growth kinetics of TiC, the change of Ti concentration within TiC from time *t*_1_ to *t*_1_ + *dt* can be approximate to the profile shown in Fig. 6a. The quantity of Ti atoms migrated to TiC is given by:

19 $$[\frac{1}{2}({c}_{{\rm{Ti}},{\rm{\min }}}^{{\rm{TiC}}}+{c}_{{\rm{Ti}},{\rm{\max }}}^{{\rm{TiC}}})({x}_{1}+d{x}_{1})-\frac{1}{2}({c}_{{\rm{Ti}},{\rm{\min }}}^{{\rm{TiC}}}+{c}_{{\rm{Ti}},{\rm{\max }}}^{{\rm{TiC}}}){x}_{1}]\cdot S=\frac{1}{2}({c}_{{\rm{Ti}},{\rm{\min }}}^{{\rm{TiC}}}+{c}_{{\rm{Ti}},{\rm{\max }}}^{{\rm{TiC}}})\,d{{\rm{x}}}_{1}\cdot S$$

S is a layer surface area. Applying the rule of Ti atom conservation at *x*_1_ and *x*_2_, the following is obtained:

20 $$({c}_{{\rm{Ti}},{\rm{\min }}}^{{{\rm{TiCu}}}_{{\rm{4}}}}-{c}_{{\rm{Ti}},{\rm{\max }}}^{{{\rm{Cu}}}_{{\rm{ss}}}})d{x}_{2}+{c}_{{\rm{Ti}},{\rm{\max }}}^{{{\rm{Cu}}}_{{\rm{ss}}}}d{x}_{1}=\frac{1}{2}({c}_{{\rm{Ti}},{\rm{\min }}}^{{\rm{TiC}}}+{c}_{{\rm{Ti}},{\rm{\max }}}^{{\rm{TiC}}})d{x}_{1}$$

Rearrange Eq. (20):

21 $$\frac{d{x}_{2}}{dt}=\frac{1}{2}\frac{({c}_{{\rm{Ti}},{\rm{\min }}}^{{\rm{TiC}}}+{c}_{{\rm{Ti}},{\rm{\max }}}^{{\rm{TiC}}}-2{c}_{{\rm{Ti}},{\rm{\max }}}^{{{\rm{Cu}}}_{{\rm{ss}}}})}{({c}_{{\rm{Ti}},{\rm{\min }}}^{{{\rm{TiCu}}}_{{\rm{4}}}}-{c}_{{\rm{Ti}},{\rm{\max }}}^{{{\rm{Cu}}}_{{\rm{ss}}}})}\frac{d{x}_{1}}{dt}$$

By integration of Eq. (21), it is obtained that

22 $${x}_{2}(t)=\frac{1}{2}\frac{({c}_{{\rm{Ti}},{\rm{\min }}}^{{\rm{TiC}}}+{c}_{{\rm{Ti}},{\rm{\max }}}^{{\rm{TiC}}}-2{c}_{{\rm{Ti}},{\rm{\max }}}^{{{\rm{Cu}}}_{{\rm{ss}}}})}{({c}_{{\rm{Ti}},{\rm{\min }}}^{{{\rm{TiCu}}}_{{\rm{4}}}}-{c}_{{\rm{Ti}},{\rm{\max }}}^{{{\rm{Cu}}}_{{\rm{ss}}}})}{x}_{1}(t)+cste$$

Incorporating Eqs (22 and 18) and the initial condition *x*_2_(*t* = 0) = 0.53 µm, the movement of TiCu_4_ boundary (*x*_2_) on the graphite side can be expressed as a function of time:

23 $${x}_{2}(t)=5.29\sqrt{1.046t+0.277}-{\rm{2.25}}\,{t}\,{\rm{in}}\,{\rm{hour}}$$

Based on Eqs (18 and 23), the thickness of Cu_ss_ (*w*_Cu_(*t*)) adjacent to TiC layer can be derived from the difference between *x*_1_(*t*) and *x*_2_(*t*):

24 $${w}_{{\rm{Cu}}}(t)={\rm{4.29}}\sqrt{1.046t+0.277}-{\rm{2.25}}\,{t}\,{\rm{in}}\,{\rm{hour}}$$

**Figure 8:**
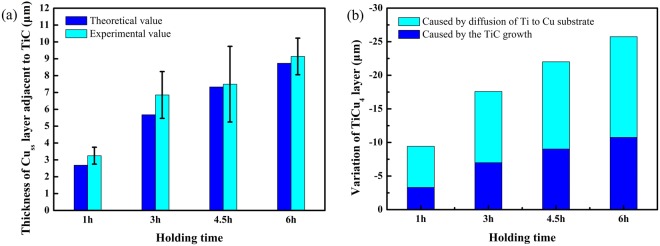
(**a**) Comparison of theoretical and experimental Cu_ss_ thickness as a function of holding time at 860 °C. (**b**) Variation of TiCu_4_ layer as a function of holding time at 860 °C.

Figure 8a shows the predicted thickness of Cu_ss_ with various holding time at 860 °C compared with the experimental data. A reasonable agreement between the predicted data and experimental data can be seen. For all the samples, the experimental data is 2%~17% higher than the calculated one in Cu_ss_ thickness. The small discrepancy is probably due to neglect of TiC particles nearby TiC layer. Though the quantity is small, the growth of these particles with large specific surface area may contribute to consuming more TiCu_4_ by solid diffusion.

Taking account of diffusion process in Cu substrate (Eq. (10)) and competing reactions with TiC (Eq. (23)), the final equation represents the variation of TiCu_4_ layer (Δ*w*) can be expressed as follows:

25 $${\rm{\Delta }}w=5.29\sqrt{1.046t+0.277}-{\rm{2.78}}+6.12\sqrt{t}\,t\,{\rm{in}}\,{\rm{hour}}$$

Figure 8b shows the predicted variation of TiCu_4_ layer as a function of time. Apparently, the quantity of TiCu_4_ consumed on diffusing in Cu substrate is prominent due to the high diffusion coefficient of Ti in Cu. The part consumed on TiC growth is around one third of the total at the beginning and it gradually increases with prolonging time. The result here also indicates that the most effective stage to eliminate TiCu_4_ is at the beginning of solid diffusion. For example, the average recession rate of TiCu_4_ layer within 1 hour (*v*_1_ = 9.43 μm/h) is more than twice of that within 6 hours (*v*_6_ = 4.29 μm/h). Considering excessive diffusion of Ti may change the property of Cu substrate (such as elastic modulus, hardness and thermal conductivity) and thicken TiC layer, rational controlling the time of solid diffusion process is not only beneficial for joining economy but also for joint property.

### Optimization of TLP bonding for copper/graphite joints

**Figure 9:**
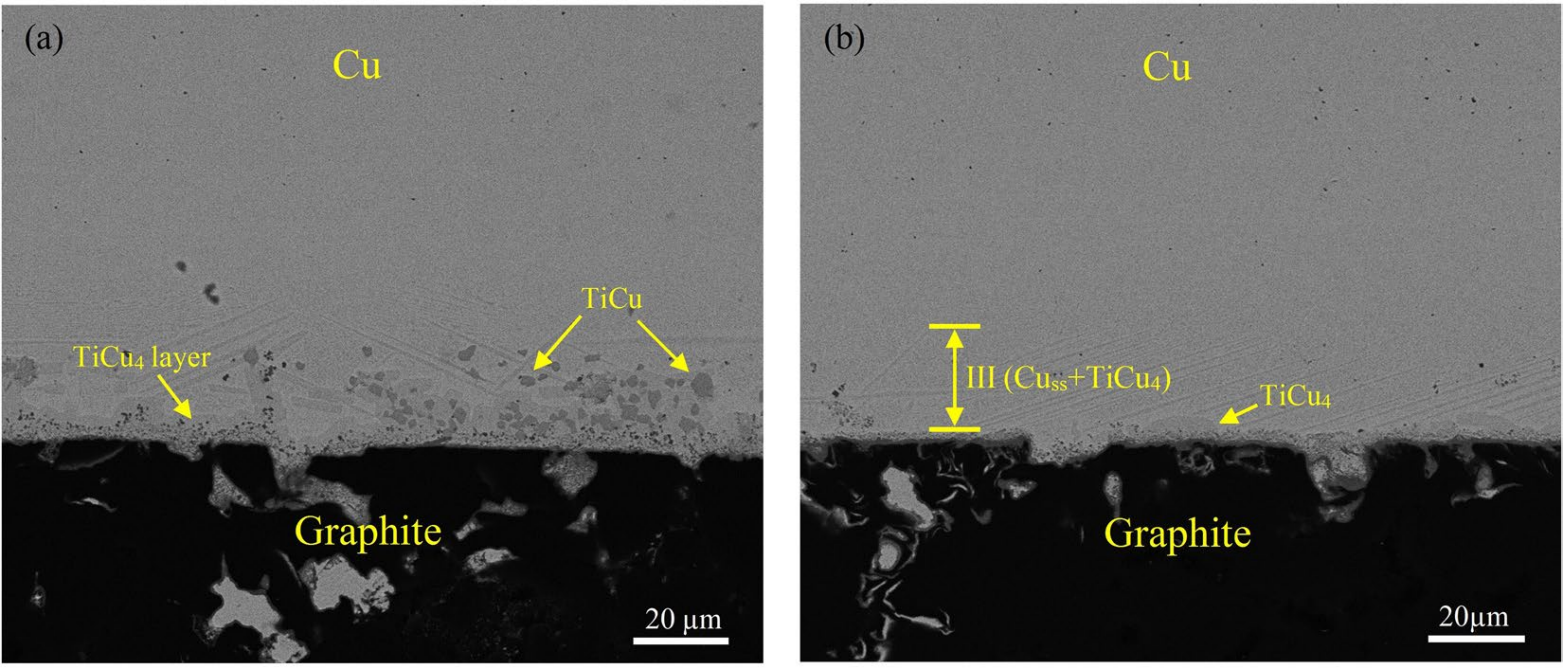
Backscattered electron micrographs of graphite/Ti (6 mm × 6 mm × 20 μm)/Cu joints prepared by (**a**) brazing at 920 °C for 5 min with pressure of 8.9 kPa and (**b**) TLP bonding (heat to 920 °C for 5 min and isothermally held at 860 °C for 1 h) with pressure of 8.9 kPa.

**Figure 10:**
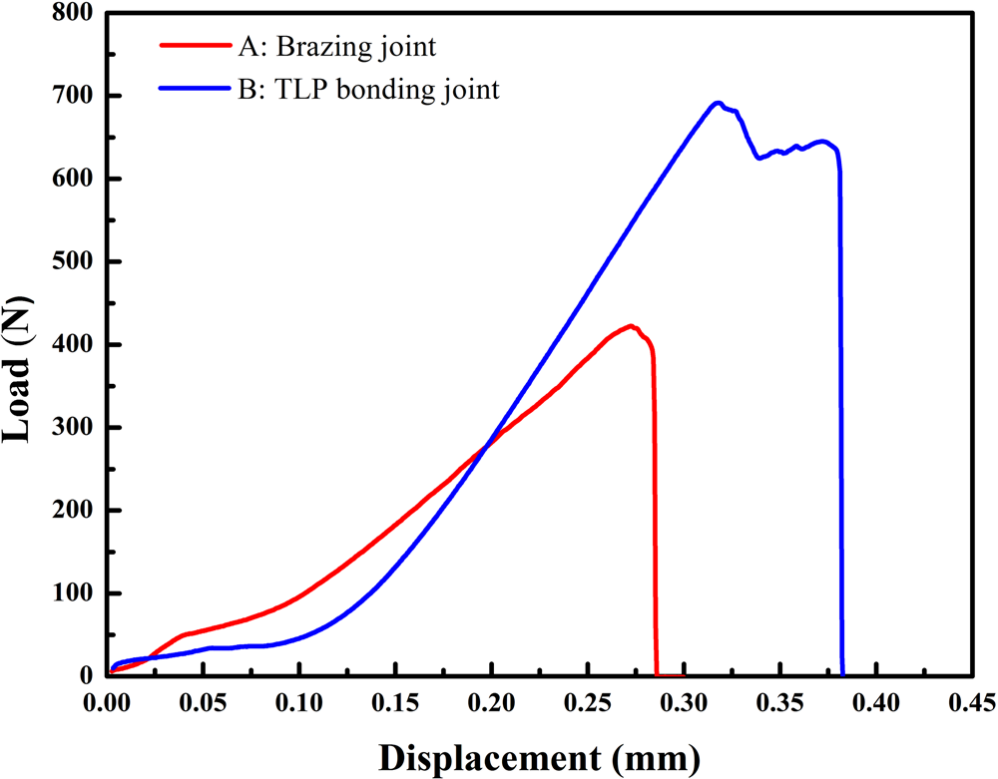
Load-displacement curves of brazing joint and optimized TLP bonding joint.

Above section reveals that solid state diffusion is an effective but time-intensive approach to eliminate residual Ti-Cu compound layer in seam. In order to maintain good performance of joints and remove the time impediment, a combination of liquid alloy extrusion and solid state diffusion is used in the optimized joining process. Figure 9a shows the interfacial microstructure of graphite/Ti/Cu joint by brazing at 920 °C for 5 min with an increased pressure of 8.9 kPa. The large scale liquid flow driven by extrusion during brazing can be evidenced by the fluctuant TiCu_4_ layer as well as the positions of TiCu compounds which have been pushed to the graphite interface (see Fig. 9a). The thickness of TiCu_4_ layer in this condition is 9.8 ± 3.4 μm thick, less than one third of the one without extrusion (as shown in Fig. 4a). According to the model proposed above, this TiCu_4_ layer can be dissipated within 1 hour by solid state diffusion. Figure 9b shows the interfacial microstructure of graphite/Ti/Cu joint by TLP bonding (brazed at 920 °C for 5 min and then solid diffusion at 860 °C for 1 h with pressure of 8.9 kPa). It is found that the residual TiCu_4_ is nearly all eliminated. In addition, the quantity of stable TiCu is also decreased significantly after solid state diffusion. This is probably due to the competing reactions between TiCu and TiC: as TiCu compounds are pushed to the graphite interface, the more stable TiC tends to grow by using Ti atoms dissolved from TiCu compounds during solid diffusion. By comparing Fig. 9a,b, it is concluded that the optimized TLP bonding with combination of liquid alloy extrusion and solid state diffusion is a feasible way to eliminate Ti-Cu compounds in a short time. The shear strengths of joints by brazing and the optimized TLP bonding are 24 ± 6 MPa and 29 ± 3 MPa, respectively. The load-displacement curves (Fig. 10) indicate both of the joints are brittle fractured. The extra stage of curve B after the maximum load is caused by the coarse fracture surface which may hinder the relative motion of the two broken parts. It’s inferred the microcracks in Ti-Cu compound layer may result in the failure of brazing joints at lower load. As for the joint by TLP bonding, though the residual stress is inevitable, defect is prevented due to the superior microstructure. So the joint has higher load bearing capability.

## Conclusion

For brazing of graphite and copper with Ti interlayer, the continuous TiCu_4_ layer is believed to be the most dangerous phase due to its cracking tendency under stress. In this work, a TLP bonding method with a diffusion process below melting point is proposed to eliminate Ti-Cu compounds in seam. A model for the degradation of TiCu_4_ layer is developed considering the simultaneous diffusion on both sides, one is the Ti diffusion in Cu substrate and the other is the Ti diffusion towards TiC layer. The latter is controlled by the growth kinetics of TiC. Based on this model, the TLP bonding is optimized to realize a Ti-Cu compound layer free joint in a short time. The shear strength of joints prepared by this method increases by 20.8% compared with that of brazing joints.

## Methods

### Sample preparation

Graphite (Beijing Electrical Carbon Co., Ltd.) and copper (Foshan Huaru Copper Co., Ltd.) are used as substrate materials to be joined in this work. The chemical compositions and mechanical properties are listed in Table 3. Graphite was cut into 7 mm × 7 mm × 2.5 mm pieces for microstructure observation and 15 mm × 10 mm × 3 mm pieces for shear tests. Copper was cut into 5 mm × 5 mm × 2.5 mm pieces. The bonding surfaces of graphite and copper were ground on SiC abrasive papers (2000#) and then polished with 1 μm diamond pastes. All samples were ultrasonically cleaned in acetone for 10 min. Joining process was carried out in a furnace with vacuum better than 1.3 × 10^−3^ Pa (oxygen partial pressure of 10^−4^ Pa). Three joining methods were carried out in this work. The first one was brazing. A 50 μm thick Ti foil was cut into 6 mm × 6 mm and sandwiched between graphite and copper with normal load of 5.9 kPa. The assembly was kept in graphite jig and heated to 920 °C at 10 °C/min, isothermally held for 10 min, then slowly cooled to room temperature at 10 °C/min. The second one was TLP bonding for Ti-Cu IMCs degradation kinetics investigation. In order to limit the flow of filler alloy, a smaller Ti foil (4 mm × 4 mm × 20 μm) was sandwiched between graphite and copper with normal load of 5.9 kPa. The assembly was heated to 920 °C at 10 °C/min, isothermally held for 5 min, then cooled to 860 °C, isothermally held for various times (0 h, 1 h, 3 h, 4.5 h and 6 h), and finally cooled to room temperature at 10 °C/min. The third one was an optimized TLP bonding. A Ti foil (6 mm × 6 mm × 20 μm) was used as filler alloy with increased load of 8.9 kPa. The assembly was heated to 920 °C at 10 °C/min, isothermally held for 5 min, then cooled to 860 °C, isothermally held for 1 h, and finally cooled to room temperature at 10 °C/min.

**Table 3:** Chemical compositions and mechanical properties of the materials.

Materials	Graphite	Copper					
Compositions (wt.%)	C ≥ 99.99	Cu + Ag	Fe	Ni	S	O	Others
		99.90	0.005	0.005	0.005	0.006	0.079
Density (g/cm^3^)	1.85	8.93					
CTE (/K)	3.9 × 10^−6^	16.5 × 10^−6^					
Elastic modulus (GPa)	10	125					
Flexural strength (MPa)	52	—					

### Characterization

The microstructure, chemistry and morphology were investigated by scanning electron microscopy (SEM, FEI Quanta 400, Eindhoven, Netherlands) equipped with an energy dispersive X-ray spectroscopy (EDS). The phases in joints were identified by an X-ray diffraction method (XRD, PANalytical Empyrean, Netherlands). The specimens for XRD test were polished layer by layer to expose the filler alloy nearby graphite interface. The thickness measurements of different phases (TiCu_4_, TiC and copper solid solution layers) were made from SEM micrographs of metallographically polished samples. These measurements were done at 10 equally spaced locations on the micrographs. The shear strength of joints at room temperature was examined using a universal testing machine (Instron-1186, Grove City, PA) with a displacement rate of 0.5 mm/min. The scheme of shear testing was reported in ref.^25^. The strength was calculated by dividing the maximum load by the surface of the joint. The tested values for each experimental condition were an average of at least six measurements.

## Data Availability

The datasets generated during and/or analysed during the current study are available from the corresponding author on reasonable request.
